# A cross-sectional molecular epidemiological study of biofilm-producing methicillin-resistant *Staphylococcus aureus*

**DOI:** 10.1097/MD.0000000000043346

**Published:** 2025-07-18

**Authors:** Nada K. Alharbi, Arwa R. Elmanakhly, Majid Alhomrani, Abdulhakeem S. Alamri, Rasha A. Mosbah, Mohamed AbdElrahman, Reham M. El-Tarabili, Fatma Alshehri, Mahmoud M. Bendary

**Affiliations:** a Department of Biology, College of Science, Princess Nourah bint Abdulrahman University, Riyadh, Saudi Arabia; b Department of Microbiology and Immunology, Faculty of Pharmacy, Modern University of Technology and Information, Cairo, Egypt; c Department of Clinical Laboratories Sciences, The Faculty of Applied Medical Science, Taif University, Taif, Saudi Arabia; d Research Center for Health Science, Deanship of Scientific Research, Taif University, Taif, Saudi Arabia; e Infection Control Unit, Zagazig University Hospital, Zagazig, Egypt; f Clinical Pharmacy Department, College of Pharmacy, Al-Mustaqbal University, Babylon, Iraq; g Department of Bacteriology, Immunology and Mycology, Faculty of Veterinary Medicine, Suez Canal University, Ismailia, Egypt; h Department of Microbiology and Immunology, Faculty of Pharmacy, Port Said University, Port Said, Egypt.

**Keywords:** biofilm, clonality, diversity, MRSA, sequence types

## Abstract

There is growing concern regarding biofilm-producing methicillin-resistant *Staphylococcus aureus* (MRSA) due to the sudden rise in infection rates and associated morbidity and mortality. Therefore, epidemiological studies, including molecular typing and correlation analysis, are essential for understanding this pathogen. This cross-sectional study investigated epidemiological factors and correlations in MRSA isolates. A total of 300 clinical samples were collected between January and March 2023 from 2 healthcare facilities, including various sample types such as sputum, blood, urine, pus, wound swabs, and other body fluids. This study employed various phenotypic and genotypic methodologies, including adherence assays using standard microtiter plates, the Congo red agar method, antimicrobial resistance and virulence profiling, and multi-locus sequence typing. Among 300 clinical samples from 2 healthcare facilities in Egypt, 94 MRSA isolates were confirmed as biofilm producers. Phylogenetic analysis revealed 8 distinct sequence types (ST8, ST80, ST239, ST15, ST22, ST113, ST398, ST984), found in surgical unit samples across both facilities. Notably, ST22-MRSA was present in all departments, indicating its widespread nature and potential for cross-departmental transmission. ST239-MRSA, the most prevalent strain (22.3%), was found in all departments except burn units. Alarmingly, 95.7% of isolates exhibited multidrug-resistant patterns. However, resistance to vancomycin and imipenem was low among biofilm-producing isolates. The high diversity of MRSA strains suggests multiple sources of infection rather than a single origin. Although most isolates were unrelated, the presence of 2 ST80 isolates in sputum samples from the same unit underscores the importance of targeted infection control within and between hospital areas. ST8-MRSA strains carrying the *vanA* gene were predominantly identified in body fluid samples, highlighting the need for regular testing in such cases. The diversity of MRSA strains across hospital departments indicates a complex infection landscape with no single source. Although certain genetic markers are linked to specific sequence types, they are not reliable indicators of MRSA clonality. These findings emphasize the need for strict infection control measures and regular testing, particularly for ST8-MRSA in body fluids.

## 1. Introduction

Methicillin-resistant *Staphylococcus aureus* (MRSA) comprises *S aureus* strains resistant to β-lactam antibiotics due to the *mecA* gene, which encodes an alternative penicillin-binding protein. The *mecA* gene is chromosomal and can transfer between strains via bacteriophages.^[1]^ MRSA is endemic in hospitals, causing nosocomial infections and spreading through contact with contaminated surfaces. It is classified into different types based on epidemiological origins. Community-associated MRSA infects healthy individuals and primarily causes skin and soft tissue infections. Healthcare-associated MRSA is linked to hospitals and invasive procedures, often exhibiting multidrug resistance.^[2]^ Livestock-associated MRSA primarily affects farm animals such as pigs and cattle and can be transmitted to humans.^[3]^

Identification of MRSA-contaminated surfaces focuses on frequently touched areas such as bed rails, doorknobs, and medical equipment due to the bacteria’s prolonged persistence.^[4]^ Transmission occurs through direct contact or respiratory droplets, while improperly sterilized medical devices such as catheters and surgical instruments can serve as reservoirs of infection.^[5]^ The emergence of multidrug-resistant (MDR) MRSA strains has led to severe infections, including vancomycin-resistant *Staphylococcus aureus* (VRSA).^[6]^ Additionally, extensively drug-resistant and pandrug-resistant MRSA strains contribute to treatment failures, posing a significant health crisis.^[7]^ The biofilm-producing phenotype of MRSA is highly pathogenic, causing bloodstream infections, osteomyelitis, and infective endocarditis, leading to increased morbidity and mortality.^[8]^ Delayed treatment can worsen outcomes, making empiric and combination antibiotic therapy crucial.^[9]^

MRSA strains are categorized into different sequence types (STs) based on genetic backgrounds, which vary by geographic region. In the United States, ST8 (USA300) is the dominant strain associated with community-acquired MRSA infections. In Europe, ST22 (EMRSA-15) and ST36 (EMRSA-16) are more common in hospital settings, whereas in Asia, ST239 has been a prevalent hospital-acquired MRSA strain.^[10,11]^ The antimicrobial resistance and virulence fitness of MRSA strains strongly correlate with their STs, which exhibit distinct resistance profiles and virulence characteristics.^[12,13]^ The evolution of MRSA clones has significantly impacted its epidemiology, with certain STs demonstrating higher toxigenic potential.^[14]^ Additionally, the presence of specific resistance and virulence genes serves as markers for identifying MRSA STs.^[15,16]^

Virulence gene profiles are closely associated with pathogenicity and disease severity, while resistance genes strongly influence treatment success. Therefore, understanding the correlations between virulence genes, antimicrobial resistance genes, and the phenotype–genotype relationship is crucial in epidemiological studies.^[17]^ However, the correlation between clinical sites, infection sources, and the fitness of virulence and antimicrobial resistance genes has not been well assessed. Moreover, these correlations have not shown consistent alignment with phylogenetic and genotypic patterns.^[18]^

To gain insights into different biofilm-producing MRSA clones, we conducted a study on MRSA isolates from 2 healthcare facilities in Egypt, using multi-locus sequence typing (MLST) to determine the prevalence of various STs and assess their antimicrobial resistance and virulence gene profiles. Our objective is to establish a comprehensive understanding of each ST’s behavior, predict their virulence and antimicrobial resistance fitness, and anticipate disease outcomes through epidemiological data analysis.

## 2. Material and methods

### 2.1. Samples collections

This report presents the findings of a cross-sectional study on MRSA isolates, aiming to investigate epidemiological factors and correlations. Between January 2023 and March 2023, a sum of 300 human clinical samples were gathered from 2 different healthcare facilities in El-Sharqia (HC1) and Port-Said (HC2), Egypt (150 clinical samples from each 1). From each health care facility, we collected sputum (25), blood (25), urine (30), pus (30), wound swab (25), different body fluid (15). In our study, we took care to collect each sample from a distinct patient, ensuring that no patient contributed multiple samples to avoid potential biases that could arise from repeated sampling from the same individual. Ensuring that each sample came from a unique patient strengthens the reliability of our data, offering a more precise depiction of the overall patient population studied. The sample collection process adhered to local and national regulations and was approved by the Research Ethical Committee of the Faculty of Pharmacy at Port-Said University (REC.PHARM.PSU) under ethical approval code REC.PHARM.PSU/2023/11. Informed consent was obtained from the participants prior to the collection of samples.^[19,20]^

Assessing infection severity was structured into 3 levels by clinical specialists: mild, moderate, and severe, by integrating clinical signs, laboratory results, imaging studies, and scoring systems. Mild infections typically presented with minor localized symptoms, slight lab marker elevations, minimal imaging findings, low scores on the Sequential Organ Failure Assessment scoring system and were managed with outpatient treatment. Moderate infections showed increased pain, redness, possible low-grade fever, moderate lab marker elevations, localized infection on imaging, intermediate scores on scoring systems, and required inpatient treatment. Severe infections were characterized by severe symptoms, high fever, tachycardia, hypotension, organ dysfunction, markedly elevated lab markers, significant imaging findings, and high scores on scoring systems, necessitating intensive care unit (ICU) admission, aggressive antibiotics, hemodynamic support, and possible surgery. Prompt recognition and treatment based on assessed severity can significantly improve outcomes and reduce complications.^[19,20]^

### 2.2. Identification of Staphylococcus aureus isolates

For the experiments, we used a brain heart infusion broth (Oxoid, UK) to enrich the bacterial growth in the tested samples. After that, nutrient agar plates (Oxoid, UK) were used to isolate the bacteria. The isolated bacteria on the nutrient agar plates were identified using standard bacteriological methods.^[21]^ This involved examining specific characteristics such as golden yellow colonies, mannitol fermentation, and β-hemolysis on nutrient mannitol salt and blood agar plates. We also examined the bacteria under a microscope to check for Gram-positive grapelike clusters and performed catalase and coagulase tests. To confirm the selected isolates, we used API-20S identification kits (BioMerieux, Marcy l’Etoile, France). Additionally, we confirmed the species of the detected isolates by conducting genetic tests using the polymerase chain reaction (PCR) technique on species-specific nuc and 16S-rRNA genes.^[22,23]^ It’s worth mentioning that we used *S aureus* ATCC6538 and *Escherichia coli* (*E coli*) ATCC25922 as positive and negative controls, respectively. These standard strains were purchased from the Animal Production Research Institute in Dokki, Giza, Egypt.

### 2.3. Characterization of biofilm-producing MRSA strains

Following the guidelines set by the Clinical Laboratory Standards Institute, we conducted methicillin resistance analysis on all the identified *S aureus* isolates. This was done in triplicate using the Kirby–Bauer disc diffusion method with oxacillin and cefoxitin screening tests. To confirm the presence of MRSA strains, we performed PCR investigations to detect the *mecA* gene.^[24]^ In addition, we identified the isolates capable of producing biofilms. This was done using a phenotypic adherence assay on a standard microtiter plate (MTP),^[25]^ and the Congo red agar (CRA) method.^[26]^ The isolates displayed diverse levels of biofilm production on CRA. Strong biofilm producers developed distinctive black colonies, moderate producers appeared as dark red to burgundy colonies, and weak producers formed light red to pink colonies. The isolates were categorized based on their biofilm production levels on MTP using the following criteria: No biofilm production (optical density (OD) ≤ ODc), weak biofilm producer (ODc < OD ≤ 2× ODc), moderate biofilm producer (2× ODc < OD ≤ 4× ODc), and strong biofilm producer (4× ODc < OD). Furthermore, we also conducted genetic tests to detect the presence of intercellular adhesion gene (*icaA*) genes.^[27]^

### 2.4. Identification of sequence types using multi-locus sequence typing

The STs of all detected biofilm-producing MRSA isolates were confirmed using the final MLST scheme, as per a previously described method. Amplification and sequencing of 7 housekeeping genes were performed, following a previously described protocol.^[28]^ These genes were guanylate kinase (*gmk*), carbamate kinase (*arcC*), glycerol kinase (*glpK*), shikimate dehydrogenase (*aroE*), phosphate acetyltransferase (*pta*), acetyl coenzyme A acetyltransferase (*yqiL*), and triosephosphate isomerase (*tpi*). All PCR assays were conducted in triplicate, with *E coli* ATCC25922 and *S aureus* ATCC25923 used as negative and positive controls respectively in all PCR runs. The alleles of the sequenced housekeeping genes were confirmed via the MLST website (https://pubmlst.org/bigsdb?db=pubmlst_saureus_seqdef), and STs were assigned according to a program available from the MLST website (http://mlst.zoo.ox.ac.uk). The STs of all biofilm-producing MRSA strains were determined using MLST. The nucleotide sequences of the housekeeping genes have been deposited in GenBank with accession numbers OR455511 to OR455604 for *arcC*, OR455605 to OR455698 for *aroE*, OR455699 to OR455792 for *glpK*, OR470096 to OR470189 for *gmk*, OR470190 to OR470283 for *pta*, OR470284 to OR470377 for *tpiA*, and OR470378 to OR470471 for *yqiL*. Following DNA amplification and sequencing, we aligned the forward and reverse sequences of all amplicons using ClustalW in the BioEdit and MEGA11 software. For phylogenetic tree construction and determination of the phylogenetic relationships, the sequences were analyzed, and a tree was drawn.^[29]^

### 2.5. Antimicrobial resistance profiles

The Kirby–Bauer disc diffusion method was used in triplicate for antimicrobial susceptibility testing of the investigated ST-biofilm-producing MRSA strains. This was carried out according to the guidelines of the Clinical and Laboratory Standards Institute (CLSI).^[30]^ Thirteen antimicrobial discs, representing all available and commonly prescribed antimicrobial classes, were used. These included chloramphenicol (C; 30 mg), oxacillin (OX; 1 mg), ceftriaxone (CRO; 30 mg), cefoxitin (FOX; 30 mg), rifampicin SV (RIF; 30 mg), tetracycline (TE; 30 mg), vancomycin (VA; 30 mg), clindamycin (DA; 2 mg), trimethoprim‐sulfamethoxazole (SXT; 1.25/23.75 mg), erythromycin (E; 15 mg), imipenem (IPM; 10 mg), ciprofloxacin (CIP; 5 mg), and gentamicin (CN; 10 mg) (Oxoid, UK). The diameters of the zones of inhibition were measured and categorized as susceptible or resistant based on the interpretive criteria in the relevant CLSI document.^[30]^ To validate the results of the disc diffusion assays, we employed the broth micro-dilution method, utilizing a 96-well MTP. Each antimicrobial drug was tested over a dilution range of 256 to 0.125 µg/mL. A well with antimicrobial drug but without microbial inoculum, and another well with microbial inoculum but without antimicrobial drug, were used as positive and negative controls, respectively. The MIC values were determined and interpreted as resistant or susceptible according to the breakpoints of the CLSI.^[30]^ Isolates resistant to 3 or more antimicrobial drugs from different classes were defined as MDR isolates.^[31,32]^

### 2.6. The resistance and virulence gene profiles

The study investigated antimicrobial resistance genes such as the vancomycin resistance gene A (*vanA*), vancomycin resistance gene B (*vanB*), erythromycin resistance gene (*ermE*), tetracycline resistance gene (*tetK*), and methicillin resistance gene (*mecA*), using PCR amplification protocols according to previous publications.^[33–37]^ A PTC-100™ programmable thermal cycler (Peltier Effect Cycling, MJ Research, Inc., UK) was used, as shown in Table S1, Supplemental Digital Content, https://links.lww.com/MD/P411.

Regarding virulence gene profiles, the virulence profiles of all ST-biofilm-producing MRSA strains were determined by detecting genes for Panton-Valentine leukocidin (*lukSF-PV*), toxic shock syndrome toxin-1 (*tst*), exfoliative toxins (*eta* and *etb*), and 9 staphylococcal enterotoxins (*sea, seb, sec, sed, see, seg, seh, sei,* and *sej*) following previously documented PCR protocols.^[38]^ In addition, genes for *S aureus* adhesin proteins, such as the clumping factor A gene (*clfA*), along with *S aureus* hemolysin proteins like the alpha hemolysin gene (*hla*), were amplified as per previously published protocols.^[39,40]^ PCR runs were conducted in triplicate using DNA from *E coli* ATCC25922 and *S aureus* ATCC6538 as negative and positive controls, respectively. These controls were sourced from the National Laboratory for Veterinary Quality Control on Poultry Production, Animal Health Research Institute, Giza, Egypt. It is important to note that all PCR runs were conducted in line with the PCR unidirectional workflow guidelines.

### 2.7. Clustering and correlation analysis

The phenotype/genotype profiles of the investigated strains were visualized using a hierarchical clustering dendrogram, and the diversity and clonality of the ST-biofilm-producing MRSA strains were assessed using a fan dendrogram in the R environment program (v. 3.6.2). This analysis was conducted following a previously described method.^[41]^ To calculate the correlation coefficients (*r-value*), the R packages *corrplot*, *heatmaply*, *Hmisc*, and *ggpubr* were utilized.^[42,43]^ In this study, correlation coefficients (*r-values*) were used to assess the associations between antimicrobial resistance, the presence of resistance and virulence gene profiles, and each ST of MRSA strains. Positive correlations were indicated by *r-values* between 0 and 1, while negative correlations were indicated by *r-values* between 0 and −1. Absolute positive and negative correlations were observed when *r-values* equaled 1 and −1, respectively. Very weak positive and negative correlations, identified by *r-values* <0.25 and >−0.25, respectively, were neglected in this study.^[44]^

Data analysis was performed using SPSS version 22 (IBM Corp., Armonk) for Windows. The chi-squared test was used to evaluate significant differences among various samples and STs. A *P*-value of <.05 was considered statistically significant. The discriminatory power (d-value) was utilized to assess the clonality and diversity of the investigated isolates. The d-value was calculated using Simpson’s index of diversity, following a previously described method.^[45]^ A d-value of 1.0 signifies that the typing method can distinguish each member of a strain population from all other members, indicating high heterogeneity and the absence of clonality. Conversely, a d-value of 0.0 indicates that all members of a strain population are of an identical type, reflecting high clonality and the absence of heterogeneity.

## 3. Results

### 3.1. Coloniality and strain variability among biofilm-producing MRSA

#### 3.1.1. Heterogeneity and coloniality of biofilm producing MRSA strains based on degree of biofilm production

Out of 300 clinical samples collected from hospitalized patients, 146 isolates of *S aureus* were recovered, representing 48.7% of the total samples. These bacteria were identified based on phenotypic characteristics, which were consistent with genetic tests targeting species-specific *nuc* and 16S-*rRNA* genes. Among the recovered isolates, 110 (75.3%) were resistant to oxacillin and cefoxitin and carried the *mecA* gene, identifying them as MRSA. Furthermore, 94 (85.4%) of the MRSA isolates demonstrated the ability to produce biofilm. Interestingly, all these isolates were classified into 3 categories: weak, moderate, and strong biofilm-producing strains. Our results showed consistent findings between the CRA and crystal violet MTP methods. The vast majority of these isolates fell into the strong biofilm-producing group, while a small number were classified into the weak and moderate biofilm-producing groups, as illustrated in Figure S1A, Supplemental Digital Content, https://links.lww.com/MD/P410. Notably, the wound swab and body fluid isolates were the most prevalent among the weak biofilm producers (Fig. S1B, Supplemental Digital Content, https://links.lww.com/MD/P410**).** Additionally, genetic analysis revealed that all these isolates harbored the *icaA* gene.

#### 3.1.2. Heterogeneity and coloniality of biofilm-producing MRSA strains based on sequence types

**Figure 1.**
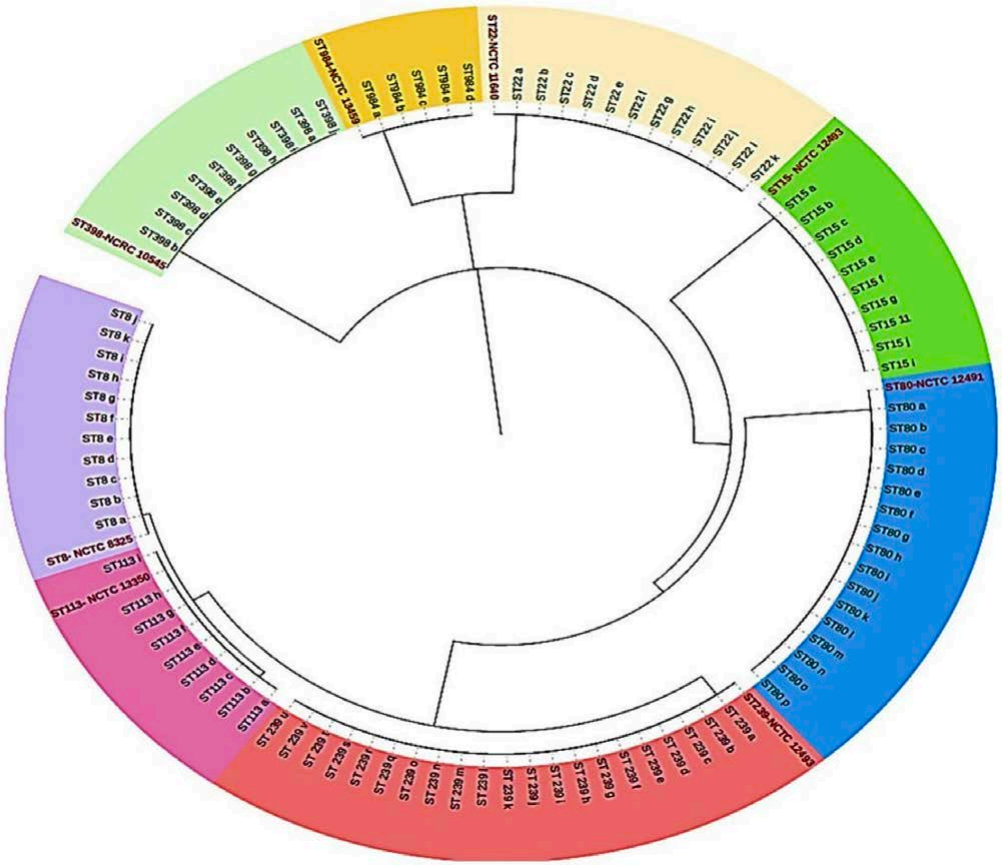
A fan dendrogram of biofilm producing MRSA Strains Based on MLST. A fan dendrogram was constructed using multilocus sequence typing (MLST) data to illustrate the phylogenetic relationships among the methicillin-resistant *Staphylococcus aureus* (MRSA) strains isolated in this study. The standard strains included in the analysis were ST8 (NCTC 8325), ST15 (NCTC 12491), ST22 (NCTC 10442), ST239 (NCTC 12493), ST80 (NCTC 12491), ST984 (NCTC 13459), ST113 (NCTC 13350) and ST398 (NCRC 10588). ST8 clade represented in purple, ST15 clade was highlighted in deep green. The light-yellow section of the dendrogram contains the ST22, the light green segment of the dendrogram shows the ST398, other sequence types like ST80, ST113, and ST239 represented in bluse, light and deep pink. MLST = multilocus sequence typing, MRSA = methicillin-resistant *Staphylococcus aureus*.

Based on MLST, all identified biofilm-producing MRSA isolates were categorized into 8 STs: ST239 (21 isolates), ST8 (11), ST80 (16), ST15 (10), ST22 (12), ST113 (9), ST398 (10), and ST984 (5). ST239 was the most common, comprising 22.3% of the biofilm-producing MRSA strains, while ST984 was the least common at 5.3%. All STs detected circulated within both healthcare facilities involved in this study, though with varying prevalence (Table 1). Overall, the fan dendrogram based on the UPGMA system illustrates the complex phylogenetic relationships and evolutionary dynamics of MRSA, providing valuable insights into its genetic diversity and epidemiology. The fan dendrogram of ST-MRSA in Figure 1 reveals significant clonality and heterogeneity among various STs. Notably, the dendrogram identified ST239 MRSA clones as high-risk variants frequently associated with severe infections and outbreaks. ST239 and ST398 exhibited high clonality, forming large, distinct clusters with multiple subtypes, indicating both genetic similarity and diversity within these groups. ST80 and ST113 also formed distinct clonal groups with internal variation, while smaller clusters, such as ST5, ST15, and ST8, exhibited less clonal expansion but still some degree of heterogeneity. The presence of numerous subtypes within larger clusters suggests genetic diversity due to mutations, horizontal gene transfers, or other evolutionary mechanisms. The clear phylogenetic separations and branching patterns indicate distinct evolutionary paths, with certain STs like ST239 and ST398 being more closely related.

**Table 1:** The prevalence of sequence types among each clinical samples and healthcare facilities.

Sample	ST239	ST8	ST15	ST22	ST80	ST113	ST398	ST984
Total	22.3	11.7	10.6	12.8	17	9.6	10.6	5.3
HC1	22	18	6	14	20	6	6	8
HC2	22.7	4.5	15.9	11.4	13.6	13.6	15.9	2.3
*P*-value	.917	.004	.034	.606	.27	.086	.034	.076
B(T)	21.4	21.4	7.1	7.1	14.3	7.1	21.4	0
B(H1)	28.6	42.8	0	0	28.6	0	0	0
B(H2)	14.3	0	14.2	14.2	0	14.2	42.8	0
*P*-value	.03	0	0	0	0	0	0	0
F(T)	28.6	42.8	0	0	0	0	14.3	14.3
F(H1)	0	75	0	0	0	0	0	25
F(H2)	66.7	0	0	0	0	0	33.3	0
*P*-value	0	0	0	0	0	0	0	0
P(T)	8	20	8	12	16	16	12	8
P(H1)	7.7	23.1	7.7	7.7	15.4	15.4	7.7	15.4
P(H2)	8.3	16.7	8.3	16.7	16.7	16.7	16.7	0
*P*-value	.88	.31	.88	.07	.82	.82	.07	0
S(T)	31.2	0	25	12.5	25	0	6.2	0
S(H1)	30	0	20	20	20	0	10	0
S(H2)	33.3	0	33.3	0	33.3	0	0	0
*P*-value	.68	0	.07	0	.07	0	0	0
U(T)	31.8	0	9.1	18.2	13.6	13.6	4.5	9.1
U(H1)	33.3	0	0	25	16.7	8.3	8.3	8.3
U(H2)	30	0	20	10	10	20	0	10
*P*-value	.68	0	0	.01	.19	.03	0	.69
W(T)	20	0	10	20	30	10	10	0
W(H1)	25	0	0	25	50	0	0	0
W(H2)	16.7	0	16.7	16.7	16.7	16.7	16.7	0
*P*-value	.2	0	0	.2	0	0	0	0

HC1 = healthcare facilities 1, HC2 = healthcare facilities 2, ST239 = biofilm producing MRSA sequence types 239, ST8 = biofilm producing MRSA sequence types 8, ST15 = biofilm producing MRSA sequence types 15, ST22 = biofilm producing MRSA sequence types 22, ST80 = biofilm producing MRSA sequence types 80, ST113 = biofilm producing MRSA sequence types 113, ST398 = biofilm producing MRSA sequence types 398, ST984 = biofilm producing MRSA sequence types 984.

#### 3.1.3. Heterogeneity and clonality of biofilm-producing MRSA strains based on antimicrobial resistance, virulence, and resistance gene profiles

**Figure 2.**
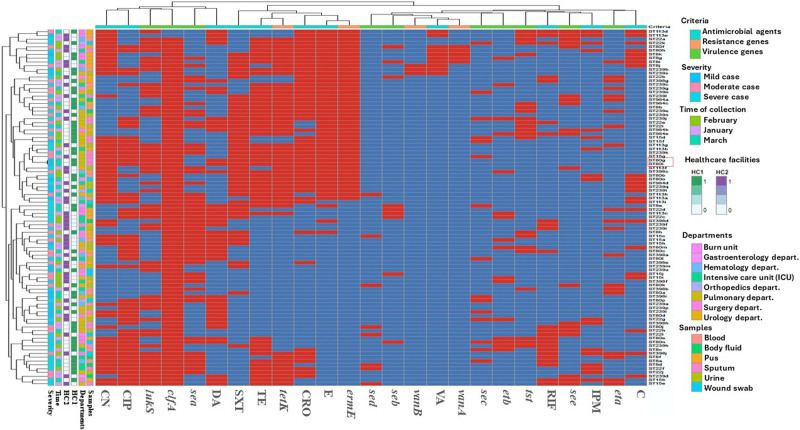
Heatmap of biofilm producing MRSA isolates based on antimicrobial agents, resistance genes, and virulence genes. This comprehensive heatmap illustrates the complex relationship between the MRSA isolates’ antimicrobial resistance profiles, the presence of specific resistance and virulence genes, the severity of the infections, the timing of sample collection, the healthcare facilities involved, the departments from which samples were collected, and the types of samples analyzed. Each row represents a unique isolate including ST239 = biofilm producing MRSA sequence types 239, ST8 = biofilm producing MRSA sequence types 8, ST15 = biofilm producing MRSA sequence types 15, ST22 = biofilm producing MRSA sequence types 22, ST80 = biofilm producing MRSA sequence types 80, ST113 = biofilm producing MRSA sequence types 113, ST398 = biofilm producing MRSA sequence types 398, ST984 = biofilm producing MRSA sequence types 984, while the columns represent different criteria, including C = chloramphenicol, OX = oxacillin, CRO = ceftriaxone, FOX = cefoxitin, RIF = rifampicin SV, TE = tetracycline, VA = vancomycin, DA = clindamycin, SXT = trimethoprim‐sulfamethoxazole, E = erythromycin, IPM = imipenem, CIP = ciprofloxacin, CN = gentamicin, *lukS* = panton-valentin leukocidin, *tst* = toxic shock syndrome toxin-1, *eta* = exfoliative toxins A, *etb* = exfoliative toxins B, *sea* = Staphylococcal enterotoxins A, *seb* = Staphylococcal enterotoxins B, *sec* = Staphylococcal enterotoxins C, *sed* = Staphylococcal enterotoxins D, *see* = Staphylococcal enterotoxins E, *seg* = Staphylococcal enterotoxins G, *seh* = Staphylococcal enterotoxins H, *sei* = Staphylococcal enterotoxins I, sej = Staphylococcal enterotoxins J, *clfA* = clumping factors A gene, *hla* = alpha hemolysin proteins, *vanA* = vancomycin resistance gene A, *vanB* = vancomycin resistance gene B, *ermE* = erythromycin resistance gene, *tetK* = tetracycline resistance gene, B (blood, 1:14), S (sputum, 1:16), F (body fluid, 1:7), U (urine, 1:22), P (pus, 1:25), and W (wound swab, 1:10), HC1 = healthcare facilities 1, HC2 = healthcare facilities 2.

**Figure 3.**
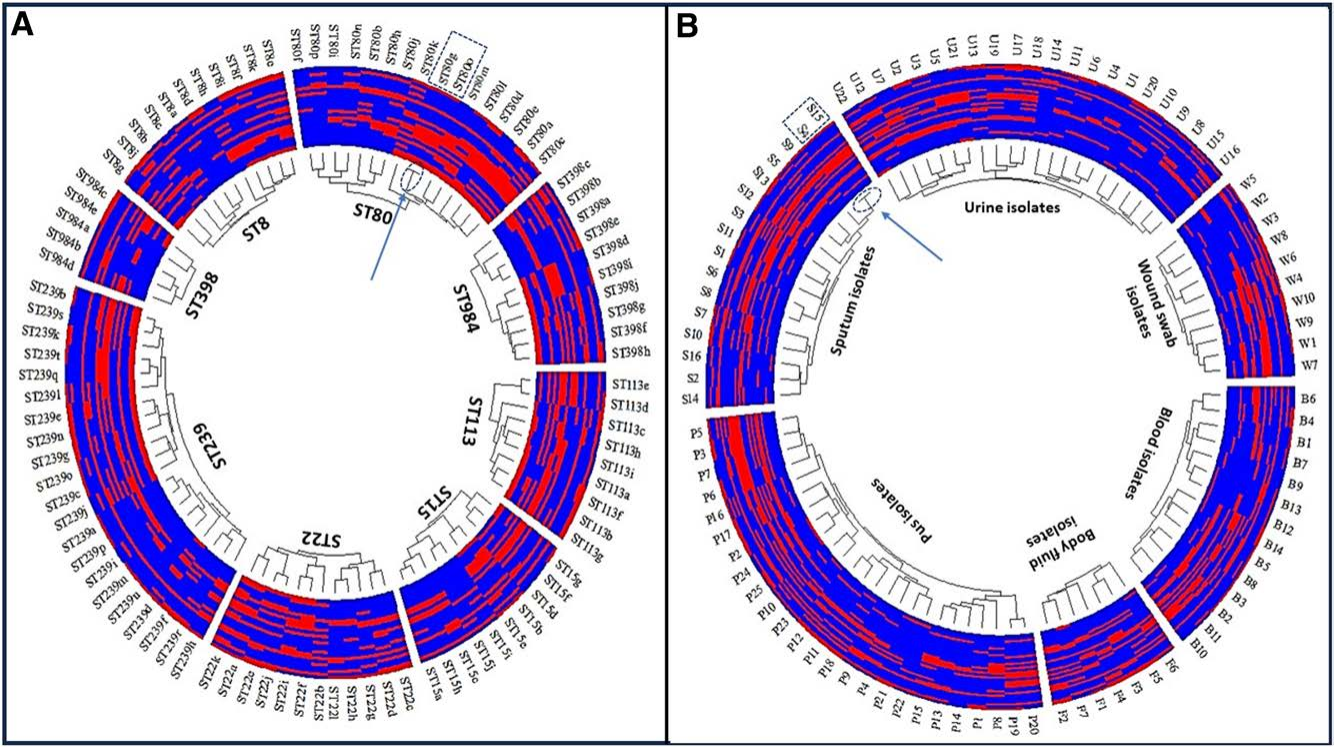
Circular dendrogram of biofilm producing MRSA within each sequence types and clinical samples. (A) Fan dendrogram showing the relatedness of biofilm producing MRSA within each sequence types, (B) fan dendrogram showing the relatedness of biofilm producing MRSA within each clinical samples, the dendrogram effectively groups the MRSA sequence types based on their genetic similarities, with a focus on their resistance patterns. Each variable (lan1 to lan29) represents a specific genetic marker or resistance gene, and the color coding provides a visual representation of whether each sequence type is resistant or sensitive to the variable in question. ST239 = biofilm producing MRSA sequence types 239 (a:u), ST8 = biofilm producing MRSA sequence types 8 (a:k), ST15 = biofilm producing MRSA sequence types 15 (a:j), ST22 = biofilm producing MRSA sequence types 22 (a:i), ST80 = biofilm producing MRSA sequence types 80 (a:p), ST113 = biofilm producing MRSA sequence types 113 (a:i), ST398 = biofilm producing MRSA sequence types 398 (a:j), ST984 = biofilm producing MRSA sequence types 984 (a:e). For panel A lan1:*lukS*, lan2:*sea*, lan3:*see*, lan4:*sec*, lan5:*seb*, lan6:*sed*, lan7: *clfA*, lan8:*hla*, lan9:*tst*, lan10:*eta*, lan11:*etb*, lan12:OX, lan13:VA, lan14:FOX, lan15:CRO, lan16:C, lan17:SXT, lan18:CN, lan19:E, lan20:DA, lan21:CIP, lan22:RIF, lan23:TE, lan24:IPM, lan25:*vanA*, lan26:*vanB*, lan27:*tetK*, lan28:*ermE*. For panel B lan1: *lukS*, lan2:*sea*, lan3:*see*, lan4:*sec*, lan5:*seb*, lan6:*sed*, lan7:*clfA*, lan8:*tst*, lan9:*eta*, lan10:*etb*, lan11:VA, lan12:CRO, lan13:C, lan14:SXT, lan15:CN, lan16:E, lan17:DA, lan18:CIP, lan19:RIF, lan20:TE, lan21:IPM, lan22:*vanA*, lan23:*vanB*, lan24:*ermE*, lan25:*tetK*, lan26:ST239, lan27:ST8, lan28:ST15, lan29:ST22, lan30:ST80, lan31:ST113, lan32:ST398, lan33: ST984. B (blood, 1:14), S (sputum, 1:16), F (body fluid, 1:7), U (urine, 1:22), P (pus, 1:25), and W (wound swab, 1:10). The red color indicates the resistance pattern or the presence of this variable, while blue color indicates the sensitivity pattern or the absence of this variable. The arrow indicates the 2 related isolates, which share identical antimicrobial resistance and virulence patterns.

The antimicrobial and virulence profiles revealed significant diversity both across different STs of MRSA clones (Fig. 2) and within individual STs (Fig. 3A). The wide array of distinct STs highlighted substantial heterogeneity and high diversity among these isolates, emphasizing the absence of clonality. On the other hand, high variability and low coloniality were observed among different clinical samples of the identified MRSA isolates (Fig. 2), indicating substantial heterogeneity within the MRSA population across different and within each clinical sample (Fig. 3B). The analysis indicated almost no clonality and absolute diversity, with a d-value close to 1, specifically 0.9997. These findings confirmed that neither the STs nor the clinical sample origins determined the clonality of MRSA clones, as the dissimilarity between isolates from different sample types and STs (Fig. 2) was similar to that among isolates from the same sample types and STs (Fig. 3A, B). Additionally, each investigated isolate in this study had a unique resistance and virulence profile. However, an exception was observed in 1 pair of isolates identified as ST 80, both recovered from sputum samples and obtained from the same healthcare facility. These 2 isolates exhibited a close genetic relationship, suggesting a possible transmission event or a common source within that facility. This finding underscores the importance of stringent infection control measures to prevent the spread of MRSA within healthcare settings.

#### 3.1.4. Heterogeneity and coloniality regarding the type of healthcare facilities, clinical samples, and case severities

Biofilm-producing MRSA isolates from HC1 exhibited higher resistance patterns than those from HC2 (Table 2). Conversely, their virulence profiles were lower (Table 3). Most items showed minimal variation across healthcare facilities, but notable exceptions were observed. Specifically, ST398 prevalence, *tst* gene presence, and resistance to chloramphenicol, rifampicin, and imipenem varied significantly (*P* < .05) across these healthcare facilities.

**Table 2:** The prevalence of antimicrobial resistance among each clinical samples and healthcare facilities.

Sample	VA	CRO	C	SXT	CN	E	DA	CIP	RIF	TE	IPM
Total	10.6	54.3	24.5	50	56.4	51.1	44.7	56.4	25.5	55.3	18.1
HC1	10	56	18	52	56	56	44	56	32	64	26
HC2	11.4	52.3	31.8	47.7	56.8	45.4	45.4	56.8	18.2	45.4	8.1
*P*-value	.762	.722	.051	.667	.94	.293	.882	.94	.051	.075	.002
B(T)	7.1	64.3	14.3	42.8	64.3	50	35.7	64.3	14.3	64.3	14.3
B(H1)	14.2	71.4	14.3	57.1	71.4	42.9	14.3	71.4	28.6	71.4	28.6
B(H2)	0	57.1	14.3	28.6	57.1	57.1	57.1	57.1	0	57.1	0
*P*-value	0	.21	1	0	.21	.16	0	.21	0	.21	0
F(T)	0	57.1	28.6	42.8	42.8	57.1	42.8	42.8	42.8	71.4	14.3
F(H1)	0	50	0	50	50	50	25	50	50	75	25
F(H2)	0	66.7	66.7	33.3	33.3	66.7	66.7	33.3	33.3	66.7	0
*P*-value	0	.12	0	.07	.07	.12	0	.07	.07	.49	0
P(T)	28	60	28	56	52	56	60	40	32	44	20
P(H1)	23.1	69.2	30.8	53.8	46.2	69.2	69.2	38.5	38.5	61.5	30.8
P(H2)	33.3	50	25	58.3	58.3	41.7	50	41.7	25	25	8.3
*P*-value	.17	.08	.44	.67	.24	.01	.08	.72	.09	0	0
S(T)	6.2	62.5	6.2	62.5	81.2	62.5	50	87.5	25	68.7	25
S(H1)	0	50	0	60	80	50	50	90	30	50	30
S(H2)	16.7	83.3	16.7	66.7	83.3	83.3	50	83.3	16.7	100	16.7
*P*-value	0	0	0	.55	.8	0	1	.61	.05	0	.05
U(T)	4.5	36.4	22.7	50	40.9	45.4	36.4	54.5	13.6	45.4	9.1
U(H1)	8.3	41.7	16.7	50	33.3	58.3	33.3	50	16.7	66.7	8.3
U(H2)	0	30	30	50	50	30	40	60	10	20	10
*P*-value	0	.17	.05	1	.07	0	.43	.34	.19	0	.69
W(T)	0	50	60	30	60	30	30	50	40	60	30
W(H1)	0	50	50	25	75	50	50	25	50	75	50
W(H2)	0	50	66.7	33.3	50	16.7	16.7	66.7	33.3	50	16.7
*P*-value	0	1	.12	.28	.03	0	0	0	.07	.03	0

C = chloramphenicol, CIP = ciprofloxacin, CN = gentamicin, CRO = ceftriaxone, DA = clindamycin, E = erythromycin, HC1 = healthcare facilities 1, HC2 = healthcare facilities 2, IPM =imipenem, RIF = rifampicin SV, SXT = trimethoprim‐sulfamethoxazole, TE = tetracycline, VA = vancomycin.

**Table 3:** The prevalence of virulence and resistance genes among each clinical samples and healthcare facilities.

Sample	*luk S*	*sea*	*see*	*sec*	*clfA*	*tst*	*eta*	*etb*	*seb*	*sed*	*vanA*	*vanB*	*ermE*	*tetK*
Total	55.3	51.1	20.2	15	97.9	23.4	21.3	17	9.6	8.5	5.3	3.2	47.9	54.3
HC1	48	46	22	22	98	30	18	12	6	10	4	4	52	58
HC2	63.3	56.8	18.2	9.1	97.7	15.9	25	22.7	12.6	6.8	6.8	2.3	43.2	50
*P*-value	.147	.287	.549	.021	.126	.435	.983	.037	.286	.069	.394	.498	.367	.441
B(T)	50	42.8	7.1	14.3	100	21.4	21.4	28.6	0	0	0	7.1	50	57.1
B(H1)	57.1	14.3	0	28.6	100	14.3	14.3	14.3	0	0	0	14.3	42.9	57.1
B(H2)	42.9	71.4	14.3	0	100	28.6	28.6	42.9	0	0	0	0	57.1	57.1
*P*-value	.16	0	0	0	1	.03	.03	0	0	0	0	0	.16	1
F(T)	71.4	71.4	28.6	14.3	100	14.3	57.1	0	14.3	14.3	0	0	28.6	85.7
F(H1)	75	75	25	25	100	0	50	0	25	25	0	0	25	100
F(H2)	66.7	66.7	33.3	0	100	33.3	66.7	0	0	0	0	0	33.3	66.7
*P*-value	.49	.49	.28	0	1	0	.12	0	0	0	0	0	.28	.01
P(T)	52	68	24	0	92	36	24	20	24	4	16	4	52	44
P(H1)	46.2	69.2	30.8	0	92.3	53.8	30.8	15.4	7.7	0	15.4	0	61.5	53.8
P(H2)	58.3	66.7	16.7	0	91.7	16.7	16.7	25	41.7	8.3	16.7	8.3	41.7	33.3
*P*-value	.24	.83	.04	0	.96	0	.04	.96	0	0	.82	0	.05	.03
S(T)	56.2	18.7	25	31.2	100	25	12.5	12.5	0	18.7	6.2	0	62.5	62.5
S(H1)	40	30	20	30	100	30	0	10	0	20	0	0	50	40
S(H2)	83.3	0	33.3	33.3	100	16.7	33.3	16.7	0	16.7	16.7	0	83.3	100
*P*-value	0	0	.07	.68	1	.05	0	.19	0	.59	0	0	0	0
U(T)	54.5	54.5	18.2	18.2	100	13.6	18.2	18.2	0	9.1	0	4.5	45.4	50
U(H1)	41.7	50	25	25	100	16.7	16.7	8.3	0	16.7	0	8.3	58.3	66.7
U(H2)	70	60	10	10	100	10	20	30	0	0	0	0	30	30
*P*-value	.01	.34	.01	.01	1	.19	.59	0	0	0	0	0	0	0
W(T)	60	50	20	30	100	20	10	10	20	10	0	0	30	50
W(H1)	50	25	25	50	100	50	0	25	25	0	0	0	50	50
W(H2)	66.7	66.7	16.7	16.7	100	0	16.7	0	16.7	16.7	0	0	16.7	50
*P*-value	.12	0	0	0	1	0	0	0	.2	0	0	0	0	1

*clfA* = clumping factors A gene, *eta* = exfoliative toxins A, *etb* = exfoliative toxins B, *ermE* = erythromycin resistance gene, *tetK* = tetracycline resistance gene, HC1 = healthcare facilities 1, HC2 = healthcare facilities 2, *sea* = Staphylococcal enterotoxins A, *seb* = Staphylococcal enterotoxins B, *sec* = Staphylococcal enterotoxins C, *sed* = Staphylococcal enterotoxins D, *see* = Staphylococcal enterotoxins E, *tst* = toxic shock syndrome toxin-1, *vanA* = vancomycin resistance gene A, *vanB* = vancomycin resistance gene B.

For urine isolates, vancomycin showed the highest sensitivity, while ciprofloxacin had the lowest. ST239 was the most frequently identified ST, with no ST8 detected. All isolates harbored the *clfA* gene, while none contained *seb* or *vanA* genes. ST15-MRSA was found only in urine samples from HC2, while ST398 was exclusive to HC1. VR isolates carrying the *vanB* gene were found solely in HC1 urine samples. ST8- and ST984-MRSA were absent in wound swab isolates, all of which were vancomycin-sensitive. None of the HC2 isolates harbored *tst* or *etb* genes, while ST15, ST113, and ST398-MRSA were found among HC2 samples (Table 3). None of the blood isolates were ST984, and none had the *seb*, *sed*, or *vanA* genes. HC1 samples contained ST15, ST22, ST113, and ST398-MRSA clones, while HC2 samples exclusively had ST80, ST8, and vancomycin-, rifampicin-, and imipenem-resistant MRSA clones. HC2 body fluid isolates were all ST8 and ST984, whereas other facilities’ isolates were mainly ST239 and ST398. All pus isolates from HC1 included ST-MRSA clones, but ST984 was absent in HC2 pus isolates. The *sec* gene couldnot be detected in any pus isolate. Sputum samples included ST239, ST15, ST22, ST80, and ST398 MRSA clones, with ST22 and ST398 exclusively from HC1. VR isolates were found only in HC2 samples (Table 1).

#### 3.1.5. Antimicrobial resistance and virulence gene profiles

Among 94 biofilm-producing MRSA strains, 90 (95.7%) exhibited MDR. Vancomycin and imipenem were most effective, with resistance rates of 10.6% and 18.1%, respectively. Gentamicin and ciprofloxacin were least effective, each with a 56.4% resistance rate. Ceftriaxone and tetracycline were least effective against ST8 and ST239. ST80 and ST15 showed the highest resistance to ciprofloxacin and gentamicin. ST22 had the highest resistance to ciprofloxacin, clindamycin, and tetracycline, while ST398 was least effective for clindamycin and ceftriaxone. ST984 was completely resistant to trimethoprim/sulfamethoxazole, tetracycline, and erythromycin. For ST113, gentamicin, erythromycin, and ceftriaxone were least effective. ST239, ST8, and ST15 were most sensitive to imipenem, while vancomycin was most effective against ST113, ST80, and ST984. ST22 showed the highest sensitivity to chloramphenicol. No VRSA strains were detected among ST15 as well as ST984, and ST22. Additionally, no imipenem-resistant strains were found among ST239, and no clindamycin-resistant strains were found among ST984 (Table 1). Genetic detection of resistance genes aligned with phenotypic resistance patterns; *ermE*, *tetK*, and *vanA* or *vanB* were only detected in strains resistant to erythromycin, tetracycline, and vancomycin (Table 3).

Approximately 96.8% of biofilm-producing MRSA strains harbored multiple virulence genes. The *hla* was found in all isolates, and the *clfA* gene in 97.9%. The *sed* and *seb* genes were least prevalent, detected in 8.5% and 9.5% of isolates, respectively, while *seg*, *seh*, *sei*, and *sej* were absent. The *lukS* gene was prevalent in ST239 and ST22, *clfA* was present in all except 22.2% of ST113, *eta* and *tst* were found in 50% of ST398 and 60% of ST984, respectively, and *etb* was found across all types with 11.1% to 20% prevalence. The ST113 and ST984 lacked *sec*, ST239, ST398, and ST984 lacked *seb* and *sed*, ST8 lacked *see*, but *sea* was present in all types with 20% to 90% prevalence (Table 3).

ST analysis revealed significant differences in *lukS*, *clfA*, and *vanA* genes, and ciprofloxacin and tetracycline resistance. Further, significant differences were found in ST8 distribution, *sea*, *seb*, *clfA*, and *tst* gene presence (Table 3) and resistance to vancomycin, chloramphenicol, clindamycin, and co-trimoxazole among clinical samples (*P* < .05; Table 2). These results underscore variability in antimicrobial resistance and virulence profiles across healthcare facilities and clinical samples.

### 3.2. Exploring pairwise correlation patterns across all studied items

#### 3.2.1. Correlation analysis between case severity and the existence of virulence genes

Our analysis revealed a significant relationship between case severity and the presence of virulence genes (including *ica* and *hla* genes) in the isolates. Specifically, 95.2% of isolates from mild cases harbored 4 or fewer virulence genes. In contrast, 83.3% of isolates from moderate cases contained 5 or fewer virulence genes. Notably, 76.7% of isolates from severe cases carried 6 or more virulence genes. Importantly, only isolates with more than 6 virulence genes were recovered from severe cases. We observed a strong positive correlation between severe cases and isolates with 6 or more virulence genes (*r-value* ≥ 0.5). Additionally, there was a strong positive correlation between mild cases and isolates with 4 virulence genes, as well as between moderate cases and isolates with 5 virulence genes (*r-value* = 0.92 each), as depicted in Figure S2, Supplemental Digital Content, https://links.lww.com/MD/P410. These findings underscore the potential role of virulence gene count in determining the severity of MRSA infections. The higher number of virulence genes in severe cases suggests a more aggressive pathogen.

#### 3.2.2. Correlation analysis between clinical samples and sequence types with antimicrobial resistance and virulence profiles

**Figure 4.**
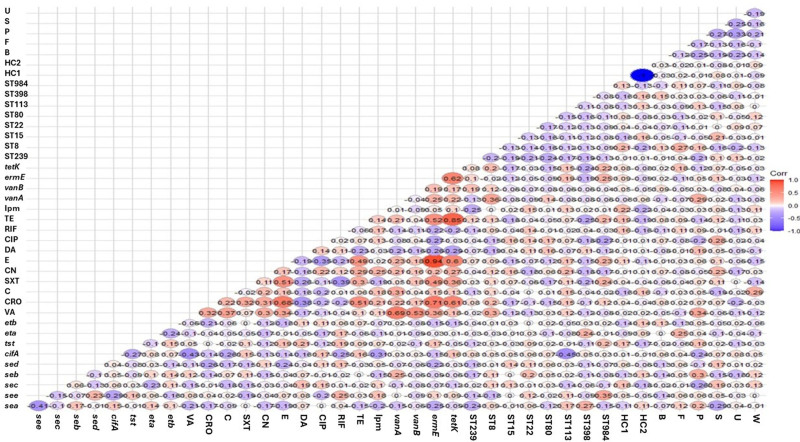
Pairwise correlation (*r*) among all investigated biofilm producing MRSA regarding to the sequence types. ST239 = biofilm producing MRSA sequence types 239, ST8 = biofilm producing MRSA sequence types 8, ST15 = biofilm producing MRSA sequence types 15, ST22 = biofilm producing MRSA sequence types 22, ST80 = biofilm producing MRSA sequence types 80, ST113 = biofilm producing MRSA sequence types 113, ST398 = biofilm producing MRSA sequence types 398, ST984 = biofilm producing MRSA sequence types 984, C = chloramphenicol, OX = oxacillin, CRO = ceftriaxone, FOX = cefoxitin, RIF = rifampicin SV, TE = tetracycline, VA = vancomycin, DA = clindamycin, SXT = trimethoprim‐sulfamethoxazole, E = erythromycin, IPM = imipenem, CIP = ciprofloxacin, CN = gentamicin, *lukS* = panton-valentin leukocidin, *tst* = toxic shock syndrome toxin-1, *eta* = exfoliative toxins A *etb* = exfoliative toxins B, *sea* = Staphylococcal enterotoxins A, *seb* = Staphylococcal enterotoxins B, *sec* = Staphylococcal enterotoxins C, *sed* = Staphylococcal enterotoxins D, *see* = Staphylococcal enterotoxins E, *seg* = Staphylococcal enterotoxins G, *seh* = Staphylococcal enterotoxins H, *sei* = Staphylococcal enterotoxins I, sej = Staphylococcal enterotoxins J, *clfA* = clumping factors A gene, *hla* = alpha hemolysin proteins, *vanA* = vancomycin resistance gene A, *vanB* = vancomycin resistance gene B, *ermE* = erythromycin resistance gene, *tetK* = tetracycline resistance gene, B = blood, S = sputum, F = body fluid, U = urine, P = pus, W = wound swab, HC1 = healthcare facilities 1, HC2 = healthcare facilities 2.

**Figure 5.**
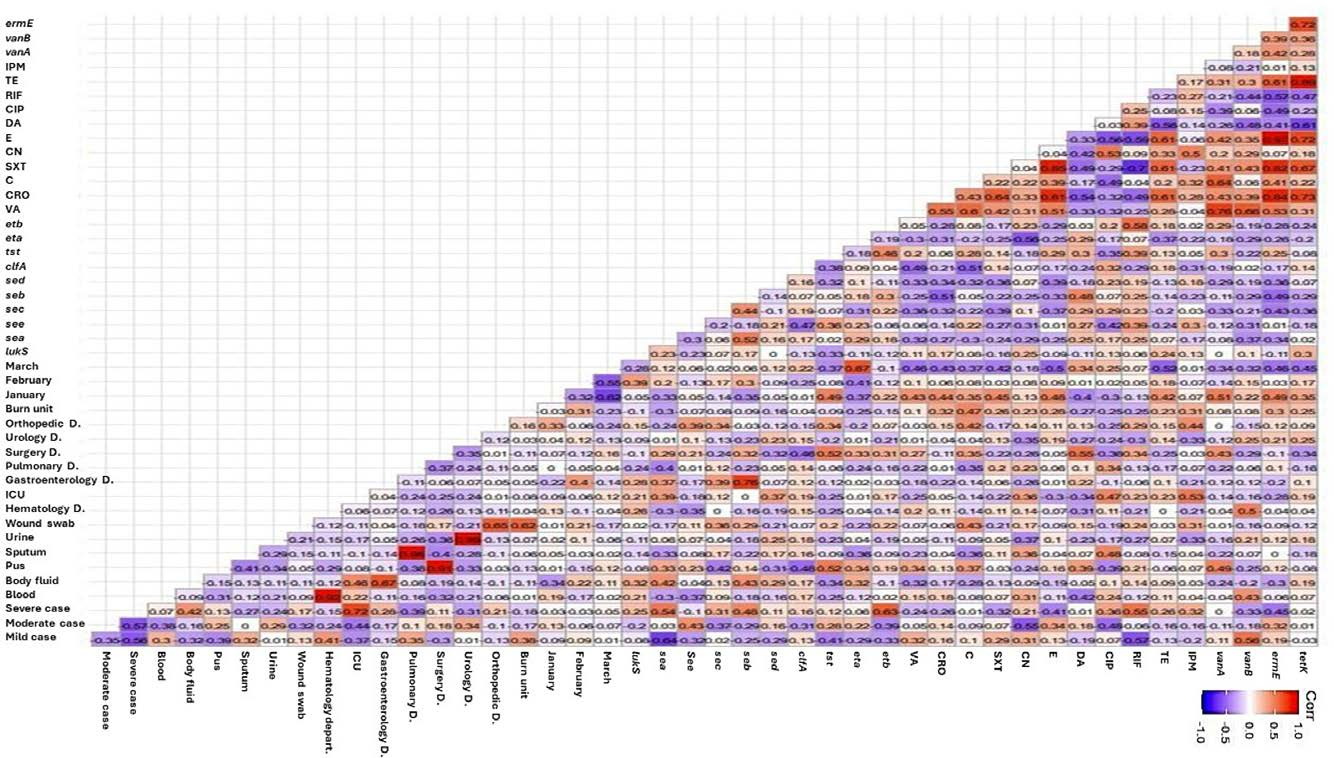
Pairwise correlation (*r*) among biofilm producing MRSA isolated in healthcare facility 1 regarding to the sample types, severity, time of collection, hospital departments. C = chloramphenicol, OX = oxacillin, CRO = ceftriaxone, FOX = cefoxitin, RIF = rifampicin SV, TE = tetracycline, VA = vancomycin, DA = clindamycin, SXT = trimethoprim‐sulfamethoxazole, E = erythromycin, IPM = imipenem, CIP = ciprofloxacin, CN = gentamicin, *lukS* = panton-valentin leukocidin, *tst* = toxic shock syndrome toxin-1, *eta* = exfoliative toxins A *etb* = exfoliative toxins B, *sea* = Staphylococcal enterotoxins A, *seb* = Staphylococcal enterotoxins B, *sec* = Staphylococcal enterotoxins C, *sed* = Staphylococcal enterotoxins D, *see* = Staphylococcal enterotoxins E, *seg* = Staphylococcal enterotoxins G, *seh* = Staphylococcal enterotoxins H, *sei* = Staphylococcal enterotoxins I, sej = Staphylococcal enterotoxins J, *clfA* = clumping factors A gene, *hla* = alpha hemolysin proteins, *vanA* = vancomycin resistance gene A, *vanB* = vancomycin resistance gene B, *ermE* = erythromycin resistance gene, *tetK* = tetracycline resistance gene, ICU = intensive care unit. Red and blue squares represent positive and negative correlations, respectively. The color legend corresponds to the correlation coefficient (*r*), with darker shades indicating stronger positive or negative correlations.

Regarding the types of clinical samples as shown in Figure 4, there was a positive correlation between ST8-MRSA clones and body fluid samples, as most isolates recovered from these samples belonged to the ST8-MRSA clone. Moreover, a positive correlation was observed between resistance to vancomycin, chloramphenicol, and ciprofloxacin with pus, wound swab, and sputum samples, respectively (*r-value* ≥ 0.25). Additionally, the presence of *seb* and *vanA* genes was positively correlated with pus samples, whereas the existence of *eta* was linked to body fluid samples. Conversely, a negative correlation was found between the presence of *sea* and *sec* genes and sputum and pus samples, respectively (*r-value* ≤ −0.25; Fig. 5).

Considering the MRSA STs, ST8-MRSA were associated with resistance to vancomycin and the presence of the *vanA* gene, while ST984 was linked with the presence of the *see* and *ermE* genes. Notably, ST398 was positively correlated with the *sea* gene. Conversely, a negative correlation was found between ST239, ST398, and ST984 and resistance to imipenem, tetracycline, and ciprofloxacin, respectively. The ST113 was negatively associated with the presence of the *cifA* gene (Fig. 5). As expected, strong correlations were found between drug resistance and the corresponding resistance genes. However, negative correlations were observed between drug resistance and the presence of virulence genes, as well as between the coexistence of some virulence genes.

#### 3.2.3. Correlation analysis between clinical samples, hospital departments and case severity with antimicrobial resistance and virulence profiles within each healthcare facility

Regarding the isolates from healthcare facility 1, the correlation analysis reveals significant associations between specific genes, clinical departments, sample types, and seasonal trends (Fig. 5). Several positive correlations were detected among antimicrobial resistances, including resistance to tetracycline with erythromycin, erythromycin with sulfamethoxazole/trimethoprim, erythromycin with ceftriaxone, erythromycin with vancomycin, and chloramphenicol with vancomycin. Additionally, positive correlations were observed between the presence of resistance genes, such as *tetK* with *ermE*, and between resistance genes with antimicrobial resistance, such as *tetK* with tetracycline as well as erythromycin and ceftriaxone, *ermE* with vancomycin. Notably, isolates from the surgical unit were associated with resistance to clindamycin and the presence of the *tst* gene, while gastroenterology isolates were linked with the existenc of *seb* gene. ICU isolates showed a positive correlation with resistance to imipenem, and pus isolates were associated with the presence of *tst* gene. Interestingly, isolates from severe cases were linked with resistance to rifampicin, the presence of *sea* and *etb* genes, and were predominantly recovered from the ICU. On the other hand, several negative correlations were observed between the presence of virulence genes and antimicrobial resistance, including existence of *eta* with gentamicin, *cifA* with chloramphenicol, and *lukS* with tetracycline as well as erythromycin.

**Figure 6.**
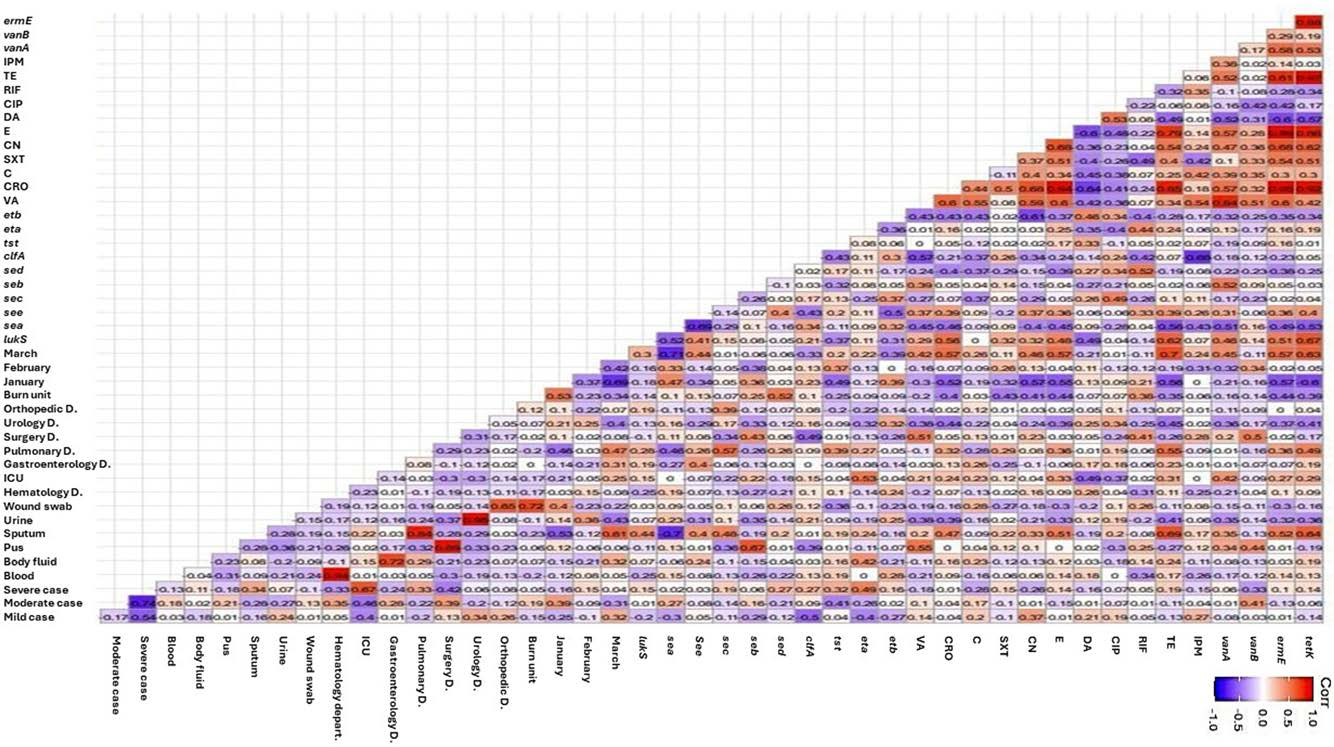
Pairwise correlation (*r*) among biofilm producing MRSA isolated in healthcare facility 2 regarding to the sample types, severity, time of collection, hospital departments. C = chloramphenicol, OX = oxacillin, CRO = ceftriaxone, FOX = cefoxitin, RIF = rifampicin SV, TE = tetracycline, VA = vancomycin, DA = clindamycin, SXT = trimethoprim‐sulfamethoxazole, E = erythromycin, IPM = imipenem, CIP = ciprofloxacin, CN = gentamicin, *lukS* = panton-valentin leukocidin, *tst* = toxic shock syndrome toxin-1, *eta* = exfoliative toxins A *etb* = exfoliative toxins B, *sea* = Staphylococcal enterotoxins A, *seb* = Staphylococcal enterotoxins B, *sec* = Staphylococcal enterotoxins C, *sed* = Staphylococcal enterotoxins D, *see* = Staphylococcal enterotoxins E, *seg* = Staphylococcal enterotoxins G, *seh* = Staphylococcal enterotoxins H, *sei* = Staphylococcal enterotoxins I, sej = Staphylococcal enterotoxins J, *clfA* = clumping factors A gene, *hla* = alpha hemolysin proteins, *vanA* = vancomycin resistance gene A, *vanB* = vancomycin resistance gene B, *ermE* = erythromycin resistance gene, *tetK* = tetracycline resistance gene, ICU = intensive care unit. Red and blue squares represent positive and negative correlations, respectively. The color legend corresponds to the correlation coefficient (*r*), with darker shades indicating stronger positive or negative correlations.

For the isolates from healthcare facility 2 in Figure 6, the correlation analysis revealed positive associations between the coexistence of resistance genes, including *ermE* with *tetK*, *vanA* with *tetK*, and *vanA* with *ermE*. Additionally, there were significant correlations between the presence of resistance genes and corresponding or non-corresponding antimicrobial resistances, such as *vanA* with vancomycin resistance as well as tetracycline resistance, and *tetK* with gentamicin resistance as well as sulfamethoxazole/trimethoprim resistance. Positive correlations were also observed among various antimicrobial resistances, including imipenem with ceftriaxone, erythromycin with ceftriaxone, erythromycin with vancomycin, and gentamicin with vancomycin. Isolates recovered from burn, surgery, pulmonary, and ICU departments were linked with the presence of *sed*, *vanB*, *sec*, and *eta* genes, respectively. Notably, sputum isolates were associated with tetracycline resistance as well as erythromycin resistance. Conversely, negative correlations were identified between antimicrobial resistances and the presence of virulence genes, including *etb* with gentamicin, *cifA* with imipenem resistance as well as vancomycin resistance, and *sea* with tetracycline resistance.

## 4. Discussion

MRSA, responsible for various global infections, affects the skin, blood, lungs, joints, eyes, and urinary tract, leading to severe conditions like bacteremia, sepsis, and death if untreated.^[46]^ It is inherently resistant to beta-lactam antibiotics,^[47]^ rendering penicillin G, oxacillin, amoxicillin, and ampicillin ineffective. Other antibiotics such as ciprofloxacin, tetracycline, erythromycin, clindamycin, and levofloxacin also show limited efficacy against MRSA.^[48–50]^ Biofilm-producing MRSA further complicates treatment due to its resistance to traditional antimicrobials.^[1]^ Biofilm-producing MRSA is a global concern in communities, hospitals, and veterinary settings.

Understanding MRSA ST distribution across healthcare facilities and hospital departments is key for effective infection control. Our study showed that all detected STs were identified in the clinical samples from each healthcare facility and surgical unit, though with varying prevalence. This genetic diversity reflects MRSA adaptation and spread within these settings. Differences in ST prevalence suggest varying epidemiological dynamics influenced by patient populations, infection control, and antibiotic usage.^[11,51]^ ST22-MRSA was found in all hospital departments, indicating its widespread nature and potential for cross-departmental transmission. Notable, ST239-MRSA was also present in all departments except burn units. The absence of ST239-MRSA in burn units might indicate specific environmental or patient-related factors that limit its survival or transmission in these areas. Previous research has demonstrated the adaptability of ST22 and ST239 strains to diverse healthcare settings, with ST22 often associated with community-onset infections and ST239 with healthcare-associated infections.^[11,52]^ Therefore, effective infection control measures, including rigorous hygiene practices and targeted antibiotic stewardship, are crucial to managing the spread of these MRSA strains within hospitals.

Interestingly, our findings indicate that STs and clinical sample origins do not drive the clonality of MRSA. MRSA are known for their genetic diversity, enabling it to adapt and thrive in diverse clinical settings. Clonality in MRSA can be influenced by various factors beyond ST or clinical sample origin, such as transmission dynamics within healthcare facilities, patient interactions, antibiotic selection pressures, and infection control practices.^[53]^ This observation is crucial for implementing effective infection control practices.^[54]^ Of note, our result highlighted an exception within the broader genetic diversity observed in MRSA isolates, emphasizing a close genetic relationship between 2 ST 80 (ST80) isolates recovered from sputum samples in the same healthcare facility. This suggests a potential transmission event or a common source within the healthcare facility. Therefore. implementing more effective infection control practices within each unit and across different units, including hand hygiene, isolation precautions, environmental cleaning, and antimicrobial stewardship, is essential to prevent the spread of MRSA and other healthcare-associated infections.^[9]^ In our study, the ST239 MRSA clones were identified as high-risk variants, frequently associated with severe infections and outbreaks. This clone has been recognized as one of the oldest epidemic MRSA strains in many countries such as Iran, India, Thailand.^[55–57]^ Similarly, in Himachal Pradesh, India, the hospital-associated multidrug resistant ST239-MRSA clone were found to be the predominant MRSA clone.^[56]^

The relationship between MRSA infection severity and virulence genes underscores their role in pathogenicity. Our findings revealed that 76.7% of isolates from severe cases had 6 or more virulence genes, with only these isolates found in severe cases. The strong positive correlation (*r-value* ≥ 0.5) suggests that more virulence genes increase disease severity, consistent with previous studies.^[58,59]^ Multiple virulence genes likely enhance the ability of MRSA to evade the immune response and cause tissue damage, aligning with prior research on the progressive impact of virulence factors.^[60,61]^ These findings highlight the importance of virulence gene profiling in predicting MRSA infection severity and guiding treatment strategies, supporting the trend toward personalized medicine.^[62]^ Comprehensive genetic surveillance in healthcare settings, along with the development of new anti-virulence therapies, is essential for monitoring virulence gene profiles. This approach can provide early warnings of highly virulent strains and support proactive measures for effective infection control.^[63]^

Understanding the link between specific MRSA strains, resistance patterns, and the types of clinical samples is essential for developing effective infection control strategies. Our findings revealed a notable association between ST8-MRSA, vancomycin resistance, and the predominance of the *vanA* gene in body fluid clinical samples. This genetic link suggests that ST8-MRSA strains may harbor genetic elements or mutations that contribute to vancomycin resistance, particularly when found in body fluid samples. In the United States and the northern region of South America, VRSA isolates were highly prevalent among ST8-MRSA clones. It has been documented that the *vanA*-containing plasmid (pBRZ01) was horizontally transferred among ST8-MRSA and ST5-MSSA clones within a short period. The genetic lineage of ST8-MRSA has a high affinity for capturing the *vanA* gene through conjugative plasmids, which supports our findings.^[64–66]^ Understanding MRSA resistance profiles, particularly in body fluids, is crucial for effective clinical practice. This knowledge aids clinicians in developing targeted treatment protocols.^[67]^ Interestingly, the *sea* gene, one of the multiple virulence genes present in the ST398-MSSA/MRSA genome, is transferred through the ϕSa3 phage. Therefore, the presence of the prophage ϕSa3 within the ST398 clone may explain the positive correlation between ST398-MRSA and the existence of the *sea* gene in our study as well as in other study.^[68]^

On the other hand, some STs showed negative correlations with antimicrobial drug resistance. In our study, resistance to imipenem was negatively correlated with the ST239-biofilm producing MRSA clone. This finding aligns with previous reports that identified imipenem as the drug of choice for treating all detected ST239-MRSA clones.^[69]^ Fortunately, the susceptibility of ST239-MRSA to imipenem reflects its effectiveness as a powerful antistaphylococcal drug.^[70]^ Tetracycline resistance, on the other hand, showed a negative correlation with the ST398-biofilm producing MRSA clone. This contradicts previous findings that reported high prevalence of tetracycline resistance among ST398-MRSA clones.^[71]^ Therefore, resistance or susceptibility to tetracycline may not be a reliable marker for identifying ST398-MRSA clones. The variation in tetracycline resistance patterns may be attributed to differences in the types of antimicrobial drugs prescribed in different geographic areas.^[72–74]^ Lastly, the ST113-MRSA clone are considered less pathogenic and lacks certain virulence genes such as *tst* and *lukS.*^[75]^ In our report, we found a negative correlation between ST113-biofilm producing MRSA and the presence of virulence genes such as *clfA.* The bottom line for this analysis, although all the announced correlations, was “none of the staphylococcal resistance or virulence genes can be used as a reliable genetic marker for identifying the ST-MRSA clones.

## 5. Conclusion

There has been a notable rise in biofilm-producing MRSA strains. Our study identified 8 STs, with ST239 being the most prevalent. A positive correlation was observed between antimicrobial resistance patterns. The high diversity and low clonality among MRSA clones highlight the need for stronger control measures. Specific STs were linked to certain genes (ST8-*vanA*, ST398-*sea*, ST984-*see/ermE*), though these genes are not reliable to be used as strain markers. The significant heterogeneity among isolates suggests multiple sources of infection.

### 5.1. Limitations

This study is limited to 2 healthcare facilities, lacks whole-genome sequencing, and uses a cross-sectional design, restricting insights into MRSA evolution and resistance mechanisms. Additionally, it does not account for community-acquired infections, patient-specific factors, and does not confirm causation in observed correlations. Therefore, future studies should include multiple healthcare facilities to better capture MRSA genetic diversity and epidemiology. Whole-genome sequencing and longitudinal designs are needed to track resistance mechanisms, gene expression, and strain evolution. Expanding research to community-acquired infections and patient-specific factors would improve understanding of transmission and pathogenesis. Additionally, functional studies on biofilm-related genes and the evaluation of infection control strategies could enhance MRSA management.

## Acknowledgments

The authors acknowledge the funding received though Princess Nourah bint Abdulrahman University Researchers Supporting Project number (PNURSP2025R153), Princess Nourah bint Abdulrahman University, Riyadh, Saudi Arabia.

## Author contributions

**Conceptualization:** Nada K. Alharbi, Reham M. El-Tarabili.

**Data curation:** Arwa R. Elmanakhly, Majid Alhomrani, Rasha A. Mosbah.

**Formal analysis:** Nada K. Alharbi, Mohamed AbdElrahman, Reham M. El-Tarabili, Mahmoud M. Bendary.

**Investigation:** Arwa R. Elmanakhly, Abdulhakeem S. Alamri.

**Methodology:** Mohamed AbdElrahman, Mahmoud M. Bendary, Fatma Alshehri.

**Resources:** Nada K. Alharbi, Fatma Alshehri.

**Software:** Fatma Alshehri, Rasha A. Mosbah.

**Writing – original draft:** Rasha A. Mosbah, Mahmoud M. Bendary.

## Supplementary Material


