# Supplementary material for: A cross-sectional molecular epidemiological study of biofilm-producing methicillin-resistant Staphylococcus aureus
Source: Medicine (Baltimore). 2025 Jul 18;104(29):e43346. doi: 10.1097/MD.0000000000043346 (PMC12282711; doi:10.1097/MD.0000000000043346)
Supplement: Supplementary file 1 [file medi-104-e43346-s001.docx]

**Table S1: The primer sequences and the PCR protocol for the analyzed resistance genes.**

| **Gene** | **primer** | **Condition** | **references** |
| --- | --- | --- | --- |
| *mecA* | F: ACTGCTATCCACCCTCAAAC  R: CTGGTGAAGTTGTAATCTGG | initial denaturation of 94°C for 3 minutes followed by denaturation at 94°C for 45 seconds, annealing at 55°C for 30 seconds, extension at 72°C for 3 minutes for 35 cycles, and final extension at 72°C for 2 minutes | Noto, M.J., Kreiswirth, B.N., Monk, A.B., Archer, G.L. (2008). Gene acquisition at the insertion site for SCCmec, the genomic island conferring methicillin resistance in Staphylococcus aureus . *J Bacteriol*. 190:1276-1283 |
| *vanA* | F: ATGAATAGAATAAAAGTTGC  R: TCACCCCTTTAACGCTAATA | an initial denaturation of 95°C for 2 minutes and then denaturation at 95°C for 1 minute, annealing at 56°C for 1 minute, extension at 72°C for 1 minute for 40 cycles, and final extension at 72°C for 5 minutes were used | Chakraborty SP, KarMahapatra S, Bal M, Roy S. Isolation and identification of vancomycin resistant Staphylococcus aureus from post operative pus sample.  *Al Ameen J Med Sci*. 2011;4:152-168. |
| *vanB* | F: ACGGAATGGGAAGCCGA  R: TGCACCCGATTTCGTTC | The vanB gene was amplified with the similar PCR reaction condition of vanA gene amplification with primer annealing temperature at 58°C for 1 minute. | Bhatt P, Sahni AK, Praharaj AK, et al. Detection of glycopeptide resistance genes in Enterococci by multiplex PCR. *Med J Armed Forces India*. 2015;71:43-47. |
| *ermC* | F: ATCTTTGAAATCGGCTCAGG  R: CAAACCCGTATTCCACGATT | PCR cycles consisted of an initial denaturation step (94°C for 5 min) followed by 30 amplification cycles (denaturation at 94°C for 60 s, annealing at 51°C [erm(A), erm(B), erm(C)] or 55°C [msr(A), mph(C)] for 60 s, and extension at 72°C for 60 s) with a final extension at 72°C for 5 min | Schlegelova J, Vlkova H, Babak V, et al. (2008). Resistance to erythro­mycin of Staphylococcus spp. isolates from the food chain. Vet Med (Praha). 53:307-314 |
| *tetK* | F: CAGCAGATCCTACTCCTT  R: TCGATAGGAACAGCAGTA | DNA amplification was carried out for 25 cycles in a final volume of 100 pi\ of reaction mixture as follows: denaturation at 94°C for 1 minute, annealing at 55°C for 1 minute, and extension at 72°C for 1 minute | Warsa UC, Nonoyama M, Ida T, et al. Detection of tet(K) and tet(M) in Staphylococcus aureus of Asian countries by the polymerase chain reaction. *J Antibiot (Tokyo)*. 1996;49(11):1127-1132. doi:10.7164/antibiotics.49.1127 |
