# Supplementary material for: A cross-sectional molecular epidemiological study of biofilm-producing methicillin-resistant Staphylococcus aureus
Source: Medicine (Baltimore). 2025 Jul 18;104(29):e43346. doi: 10.1097/MD.0000000000043346 (PMC12282711; doi:10.1097/MD.0000000000043346)
Supplement: Supplementary file 2 [file medi-104-e43346-s002.docx]

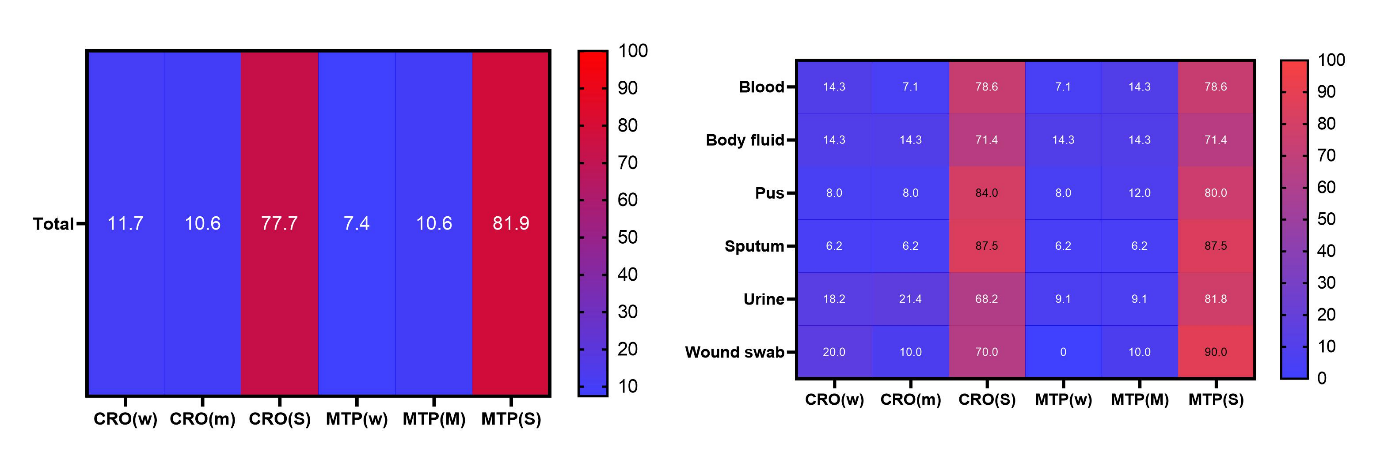


**Fig. S1:** **Heatmap illustrating the biofilm production of MRSA strains using Congo Red Agar and Microtiter Plate assays.**

CRO(w): weak biofilm producing isolates on Congo Red Agar, CRO(m): moderate biofilm producing isolates on Congo Red Agar, CRO(s): strong biofilm producing isolates on Congo Red Agar, MTP(w): weak biofilm producing isolates by microtiter plate method, MTP(m): moderate biofilm producing isolates by microtiter plate method, MTP(s): strong biofilm producing isolates by microtiter plate method. The color gradient represents values where smaller values are indicated by blue and increase in intensity towards red.


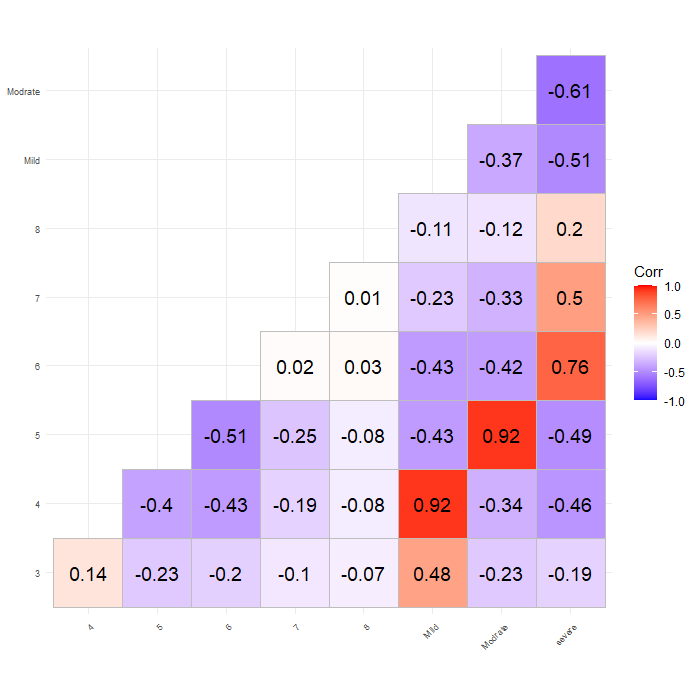


**Fig. S2: Pairwise correlation (r) between the case severity and the existence of virulence genes**

Red and blue squares represent positive and negative correlations, respectively. The color legend corresponds to the correlation coefficient (r), with darker shades indicating stronger positive or negative correlations.
